# Commissioner, clinician, and patient experiences of a pre‐surgical health optimisation programme – a qualitative study

**DOI:** 10.1186/s12913-021-06434-z

**Published:** 2021-05-01

**Authors:** Joanna McLaughlin, Cecily Palmer, Sabi Redwood, Ruth Kipping, Lucie Owens, Rebecca Reynolds, Lauren J Scott, Elizabeth M Hill, Jenny L Donovan, Russell Jago, Amanda Owen-Smith

**Affiliations:** 1 Population health Sciences, Bristol Medical School, University of Bristol, Bristol, UK; 2 Bath and North East Somerset Council , Bath , UK; 3 National Institute for Health Research Applied Research Collaboration West (NIHR ARC West), University Hospitals Bristol and Weston NHS Foundation Trust, Bristol, UK; 4 Bath and North East Somerset Clinical Commissioning Group, Bath, UK; 5 Centre for Exercise Nutrition & health Sciences, School for Policy Studies, University of Bristol, Bristol, UK

**Keywords:** Health optimisation, Prehabilitation, Elective surgery, Behavioural change, Obesity, Smoking

## Abstract

**Background:**

Health optimisation programmes are an increasingly popular policy intervention that aim to support patients to lose weight or stop smoking ahead of surgery. There is little evidence about their impact and the experience of their use. The aim of this study was to investigate the experiences and perspectives of commissioners, clinicians and patients involved in a locality’s health optimisation programme in the United Kingdom. The programme alters access to elective orthopaedic surgery for patients who smoke or are obese (body mass index ≥ 30 kg/m^2^), diverting them to a 12-week programme of behavioural change interventions prior to assessment for surgical referral.

**Methods:**

A thematic analysis of semi-structured interviews (*n* = 20) with National Health Service and Local Authority commissioners and planners, healthcare professionals, and patients using the pathway.

**Results:**

Health optimisation was broadly acceptable to professionals and patients in our sample and offered a chance to trigger both short term pre-surgical weight loss/smoking cessation and longer-term sustained changes to lifestyle intentions post-surgery. Communicating the nature and purpose of the programme to patients was challenging and consequently the quality of the explanation received and understanding gained by patients was generally low. Insight into the successful implementation of health optimisation for the hip and knee pathway, but failure in roll-out to other surgical specialities, suggests placement of health optimisation interventions into the ‘usual waiting time’ for surgical referral may be of greatest acceptability to professionals and patients.

**Conclusions:**

Patients and professionals supported the continuation of health optimisation in this context and recognised likely health and wellbeing benefits for a majority of patients. However, the clinicians’ communication to patients about health optimisation needs to improve to prepare patients and optimise their engagement.

## Background

### Policy context

Pathways to selected surgical interventions are being redesigned with increased use of ‘health optimisation’ (HO) interventions or ‘prehabilitation’ across health systems internationally, including the United Kingdom’s National Health Service (NHS) [1]. Most commonly, HO policies are applied to hip and knee elective surgery pathways. Their purpose is to encourage eligible patients to lose weight, stop smoking and increase fitness before surgery. The intended outcomes include a reduction in surgical procedures, improved safety, outcomes and recovery from surgery, and to trigger lasting lifestyle change [2].

Health optimisation presents an interplay between rationing for improved resource allocation and health improvement. Existing literature highlights the ethical concerns around imposing thresholds for surgery and the rationing of healthcare based on factors commonly related to lifestyle choices, such as body mass index (BMI) [3, 4]. The Royal College of Surgeons has stated that all commissioning policies should be based on clinical need and not factors such as smoking status or weight [5]. Despite this guidance, over half of England’s clinical commissioning groups (CCGs) have such policies that ration access to joint replacement [6, 7].

The socioeconomic patterning of smoking and obesity means that HO policies have the potential to exacerbate health inequities depending on their sensitivity to the needs of marginalised groups in society. Given that 62 % of adults in England are overweight, 14 % are smokers [8] and over half of CCGs have HO policies [9], thousands of NHS patients are already directly affected. The COVID-19 pandemic led to the suspension of much elective surgery worldwide, and as a result the number of patients awaiting such surgery has further increased [10]. Nevertheless, effective pre-surgical health optimisation could be of significant benefit to healthcare systems if the hypothesised reduced need for surgery and improved surgery outcomes are shown to be true [1].

Despite many HO policies having been in operation for several years, evaluations of their impact have not been published and evidence for their effectiveness remains unclear [2, 3]. In addition, the range of these commissioning policies suggests there is uncertainty over appropriate eligibility thresholds and policy content.

### Research context

A clinical commissioning group in South West England introduced an ambitious ‘Getting Fit for Surgery’ policy in late 2017, aimed at altering access to elective surgery for patients who smoke or are obese (body mass index (BMI) ≥ 30 kg/m^2^). Unless flagged as urgent or meeting exclusion criteria, on referral from their general practitioner (GP) these patients are diverted to a health optimisation programme for 12 weeks to be offered behaviour change interventions via referral to the ‘Healthy Lifestyles Service‘ prior to being re-assessed for appropriateness for surgical referral.

**Fig. 1:**
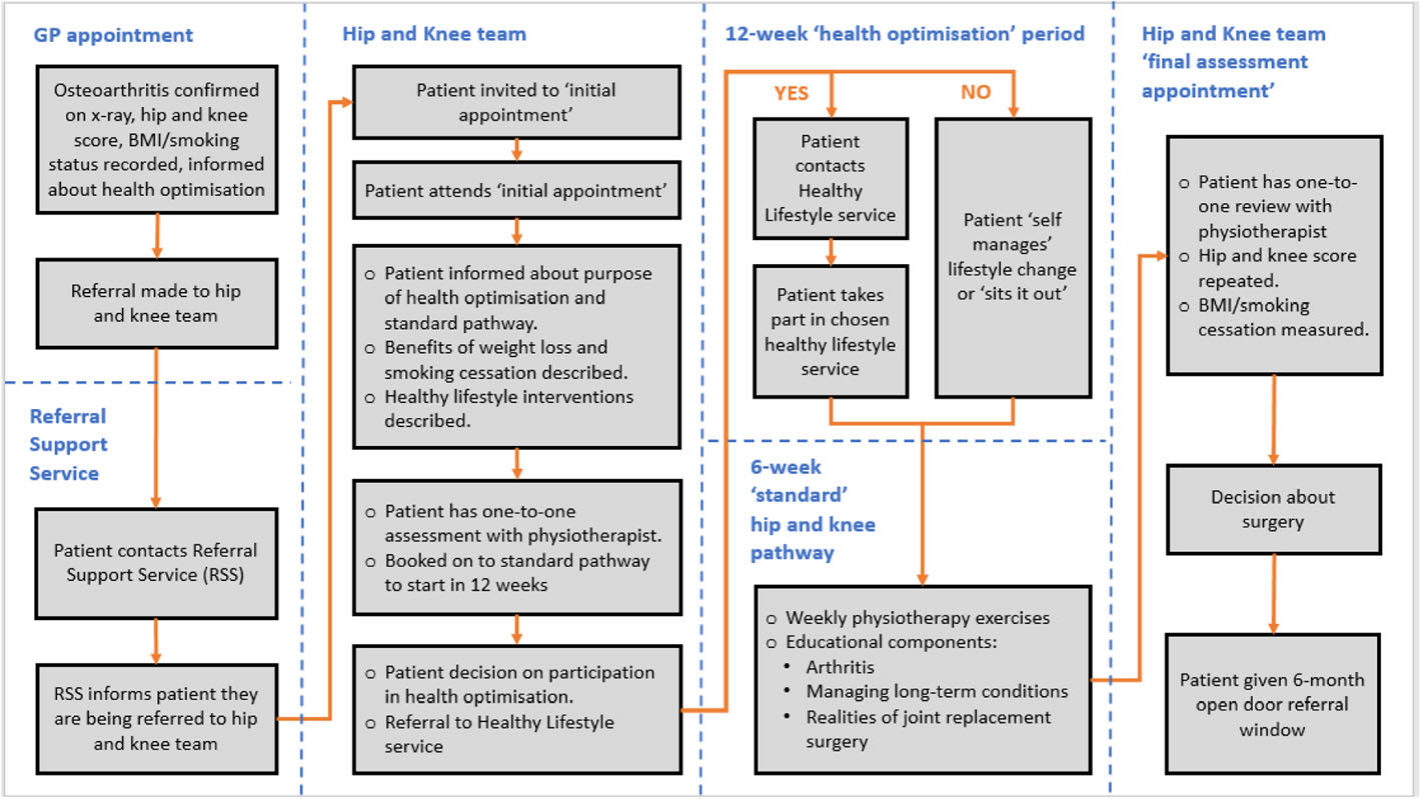
The hip and knee pathway for health optimisation and non-health optimisation* patients. *Note. Non-health optimisation patients proceed immediately from the initial hip and knee team appointment to the 6-week ‘standard’ hip and knee pathway.

The policy has been implemented for patients needing referral for hip or knee osteoarthritis. Figure 1 shows a flowchart of the patient pathway.

The introduction of the policy provided an opportunity to complete a feasibility study of the evaluation of such a policy. Evaluation could identify generalisable implications for other settings considering the introduction of similar policies and their evaluations.

A separate paper reports on the feasibility of policy evaluation with use of the quantitative findings of the policy evaluation [11] and gives detail on the flow of patients through the pathway and the patient characteristics.

This qualitative study aimed to investigate the purpose of the HO strategy from the perspective of those planning and commissioning the pathway. It also aimed to investigate the perspectives of GPs and physiotherapists involved in implementing the new pathway, and patients experiencing the pathway.

## Methods

Semi-structured interviews with clinical and public health commissioners and planners, healthcare professionals, and patients using the hip and knee health optimisation pathway.

### Sampling and recruitment

The research team identified categories of the main professional groups with influence on the policy creation, implementation and with experience of in-person delivery of the pathway. Clinicians GPs and members of the hip and knee physiotherapist led team), Healthy Lifestyles team members, and CCG and public health commissioners involved in the planning and delivery of the pathway were purposefully sampled for invitation to participate. Twenty potential participants were identified from membership of the HO policy steering group and professionals involved in delivering relevant patient services. An invitation explaining the purpose of the study was sent by email from the research team, accompanied by an information sheet, and was followed up with a single reminder if no response had been received after two weeks.

Purposive sampling of patients was undertaken through collaboration with the hip and knee team who identified patients on the HO pathway. They aimed to include a balance of gender, clinical characteristics (Oxford score, BMI, smoking status), engagement with the pathway, and surgical outcome. Recruitment was through invitation letter sent by the hip and knee team. Forty patient invitations were sent. Replies were made directly to the research team who had no prior access to identifiable information and therefore non-responders in the patient group could not be followed up.

### Data collection

Interviews were carried out (by CP or JM) either face-to-face at a community hospital or by telephone. Before the interview, participants had the opportunity to ask questions, before providing written consent to participation, audio recording and publication of anonymised data extracts. Topic guides for semi-structured interviews were developed by the research team and informed by patient and public involvement (PPI). The key areas of enquiry are described in Table 1.

**Table 1:** Key areas of enquiry in interviews by participant group

Participant group	Key areas of enquiry in topic guide
Patients	• understanding of the decision-making / referral process • experiences of participating in the health optimisation pathway; impact on personal health / well-being / immediate family / socio-economic context • expectations of, and decision-making about, ongoing care
Managerial	• how the plans for the health optimsation pathway were developed • anticipated risks and benefits of the scheme • expected impacts of the scheme in the future
Providers	• experiences of using the pathway; delivering interventions and engaging with patients • patient reactions to health optimisation and the impact on patient well-being • future direction; views on extension of health optimisation from hip & knee services to broader elective surgeries

### Patient and public involvement

The overall aims and objectives of the study were discussed at the National Institute for Health Research (NIHR) Applied Research Collaboration (ARC) West Health Systems PPI Panel meeting in February 2019. The discussion’s main aim was to identify which of the listed objectives were the topmost priority, and which stakeholder groups were the most important to engage with. The priorities raised were used to inform the interview topic guides. Feedback was also sought on key terminology used in patient information.

### Data analysis

Interviews were audio-recorded and fully transcribed. The transcripts were anonymised and checked for accuracy against the recordings by the interviewers. Data were analysed thematically using methods of coding and constant comparison to identify and inter-relate emerging themes These methods were consistent with a constant comparison approach, as initially explicated in the development of grounded theory by Glaser and Strauss [12]. The analysis thus took an inductive approach. All transcripts were read and reread to identify the major themes before open coding using qualitative data analysis software (NVIVO) was applied to blocks of text. Each interviewer (JM and CP) coded the interviews they had conducted. Six interviews were double coded/analysed separately by the other interviewer or by co-author AOS to enhance analytic rigour; differences in interpretation were discussed until agreement was reached. Data analysis ran in parallel with sampling and data collection so that emerging themes could be followed up and synthesised. Negative cases, where informants held divergent views or experiences did not follow the standard course of events, were re-analysed to gain further insights. Themes emerging from the data were written up as detailed descriptive accounts to facilitate the inter-relation of emergent themes and compare findings across participant groups and between individual participants prior to the final analysis and presentation of the data.

Ethics.

 This study was reviewed by the North West/Liverpool East NHS ethics committee (IRAS no: 258,508), and written informed consent was received for all participants. All methods were performed in accordance with the guidelines and regulations of the sponsor organisation.

## Results

The final sample comprised 20 participants. The characteristics and distribution of patient and professional informants are shown in Table 2. All the patients were white British and were aged between 50 and 76 years. The interviews lasted around 60 min (range 30 to 90 min).

**Table 2:** Characteristics of the interview participants

Role	Number	Characteristics
Managerial	4	Clinical commissioning group or local authority commissioners and policy makers
Provider	9	Hip and Knee team, Healthy Lifestyle Service, GPs, Referral Support Service
Patient	7	• 5 males, 2 females. • All patients had obesity; one was also a smoker. • 4 accepted Healthy Lifestyle Service support referral, 3 declined • 5 referred for surgery, 2 referred for conservative management

Themes identified in the analysis were organised into four major categories: the organisation of the HO pathway, experiences of the pathway in practice, experiences of communicating about the pathway, and views on the future of the pathway. Insights into how health optimisation was successfully implemented as part of the hip and knee pathway, but failed in wider roll-out, are also reported. Below we discuss each of these aspects with use of illustrative quotes. Quotations were selected from a range of informants to illustrate the themes. Ellipses (…) are used to denote omitted text.

### 1. Organisation of the health optimisation pathway

The main themes addressed in this category relate to participant views on the aims of the pathway and issues concerning its timing regarding expected date of surgery.

#### Aims and purpose of the pathway

Professional and patient participants perceived that the reasons for the introduction of the health optimisation programme included: improved surgical outcomes and safety, symptom improvement and reduced need for surgery, and a long-term opportunity to improve health by enabling personal responsibility for health-related behaviour.

Several professional participants reported that an initial key driver for the HO policy development was the potential for financial savings through a reduction in surgical treatments.

*“Getting Fit for Surgery (…) could be an area that we could look at to maybe see whether there would be potentially some efficiency savings.”* (Managerial M2).

However, as further consideration was given to the evidence and likely outcomes, several participants reported that the main impetus for policy introduction altered to that of health improvement and reduction of inequalities, without expectations of financial benefit.

*“Using that opportunity to get people to have that teachable moment to think, oh actually this is an opportunity for me to take personal responsibility, do something about my health.”* (Managerial M2).

*“I then changed my mind (…) there was a potential for [health optimisation] to reduce inequalities, by increasing the numbers of people who stopped smoking and lost weight.”* (Managerial M4).

Where patient participants reflected on the purpose of the HO pathway, most thought that a major aim of the pathway was to bring about symptom improvement and improved clinical outcomes; six patients perceived a clear association between weight loss or smoking cessation and improved outcomes after surgery including quicker recovery, better healing, fewer infections or reduced complications.

*“being seriously overweight for any operation is not a good thing and I think most surgeons would prefer to see people with a sensible weight before doing a major operation.”* (Patient P3).

Some patients perceived that cost saving and promoting self-responsibility for health were also commissioner motives, though felt that these need not be at odds with a positive policy impact.

*“I don’t think it’s unreasonable for the NHS to refuse operations to people who aren’t prepared to self-help.”* (Patient P5).

### Timing of the health optimisation period

While there was widespread professional buy-in to the aims of the policy, its organisation and delivery by way of a 12-week mandatory window for HO raised concerns for some professional participants about the potential perception of a ‘two-tier’ or ‘admonishing’ service due to the longer timeframe before being referred to see a surgeon.

*“I had a real concern around inequalities (…) those who already were struggling in life with various things (…) it was ‘penalising’”* (Managerial M4).

Those who delivered the pathway were discomforted by having to inform patients that they would undergo a delay and some concerns were raised that physical symptoms could worsen during the 12-weeks.

*“a lot of anxiety (…) how annoyed people might be at being told ‘no, you have to wait 12 weeks before you even start your exercise programme’”* (Provider Pr10).

Consequently, four professionals expressed a preference that health optimisation be offered during existing waiting time for surgical referral rather than in an additional window of 12 weeks.

*“I wanted it to run so that if someone was overweight or if they were smokers, yes, they get referred to Slimming World but then they start their [physiotherapy] exercise at the same time.”* (Provider Pr6).

In addition, four patients stated that the proximity of the time period for lifestyle change to the expected date for surgery was likely to have an important impact on patient motivation and potential to benefit.

*“you could be waiting five months; ‘Oh, what’s the point? I’ll go and watch television and have a couple of cans of beer.’”* (Patient P2).

### 2. Communicating about the health optimisation pathway

#### Consistency and ownership

Professionals involved in the delivery of the pathway were concerned that many patients attended the first appointment of the health optimisation pathway without awareness or understanding of it. Secondary care professionals reported having experienced anger from patients on hearing of the additional 12-week window.

*“The GP hadn’t necessarily informed them of exactly what was gonna happen. (…) and people did feel very angry, at times, angry that they weren’t going straight to the surgeon.”* (Provider Pr8).

Professionals desired consistency in how the pathway is communicated to patients. They perceived that acceptability would be increased if all clinicians, particularly the GP in the initial appointment, took ownership and expressed a positive attitude toward the pathway.

*“It’d be good if the GP communicated the health optimisation part as well. Even if it was just the basic, you know, ‘there’s this part of the pathway’. (…) Just kind of outlining this is the pathway, this is what it involves, would make it a lot easier.”* (Provider P11).

Two GP participants reported that conversations about HO could be uncomfortable due to both raising the issue of weight, and more importantly having to inform patients that they would undergo a 12-week delay before being able to see a surgeon. The short timeframe of the GP consultation within which to navigate these challenging conversations was also emphasised.

*“I think that in a pressured system (…) where these conversations take longer (…) so we have to create time for (…) what can be quite challenging conversations with patients who may arrive at the expectation of having an operation”* (Managerial M7).

As time went on, GP participants described gains in skill and confidence in communicating the benefits of the HO pathway and in introducing it to their patients. Hip and knee team members described that they addressed poor patient awareness of HO through clear communication of the evidenced value of weight loss and/or smoking cessation in terms of symptom management, post-operative benefits and longer-term recovery success, and perceived that this facilitated patient ‘buy-in’ to HO.

*“(…) [the providers] were able to provide some support (…) how to sell it and get alongside [patients] and enable them to have those conversations in a more beneficial way for both parties.”* (Managerial M5).

#### Patient views

No patients reported any difficulty with their GP introducing the requirements of the pathway. Four patients stated that they think the introduction of health optimisation in an initial GP appointment is welcome, appropriate and often not surprising.

*“have the courage to say ‘Look, you’re overweight, you need to lose it. This smoking business you need to stop doing it.’ Doctors perhaps need to toughen up. If they offend people, well perhaps sometimes you have to do it to make them think about what’s been said to them.”* (Patient P6).

Patients highlighted that the pathway felt as if it was focused on surgical preparation with some describing a perception that surgery was a preferred outcome. However, the outcome most desired by patients was improved mobility and decreased pain, with two patients specifically aiming to avoid surgery.

*“I think, you know I’d avoid saying about ‘the pathway to surgery’ (…) ‘cause there’s the fact that I lost a bit of weight and the fact that my pain reduced quite substantially meant that I didn’t need the operation.”* (Patient P7).

### 3. Experience of using the health optimisation pathway

#### Reasons for engagement and non-engagement with the support offered

Patient recollections of their response to the offer of support for weight loss or smoking cessation varied; some described eager acceptance of help.

*“So, when she [GP] mentioned the overweight and I said ‘yes, I know I’m overweight and I really do need somebody to talk to me seriously about it’. That fitted in nicely with what I wanted to do.”* (Patient P6).

Others described neutral engagement only due to the mandatory 12-week window, with choices made based on familiarity or practicality.

*“Bit puzzled at the time. Actually, my daughter also goes to [Slimming World] at the same place (…) So, I started going and found it very successful.”* (Patient P3).

The predominant reason given by those patients who chose to decline support was that they were already well-informed about healthy lifestyle advice and had undergone many past failed attempts at change.

*“I would try to lose weight, but I did finish up (…) saying, ‘Well I’m living on rabbit food here and I’m not losing any weight’ (…) ‘Not doing this anymore’.”* (Patient P5).

#### Weight loss experience and outcomes

Those patients who took up support had all attended group sessions and described these as acceptable. Several had achieved significant weight loss.

*“in the first (…) ten or 11 days, I lost eight pounds and won a certificate and then went on and lost another nearly a stone”* (Patient P7).

Impact on diet and lifestyle extended to family members in two cases.

*“I’m not eating things I used to eat, and he doesn’t now (…) So he’s doing it by default really. He’s lost a stone as well so it’s good for both of us isn’t it?”* (Patient P6).

However, none of the patients interviewed reported deterioration in their physical symptoms over the HO period, though one felt that the additional length in the pathway and uncertainty over date of surgery had been a source of stress.

*“not knowing and having no time factor on when it’s [surgery] going to happen is stressful.“* (Patient P2).

Two patients described a lack of support for weight maintenance after the 12-week HO programme had finished and reported that they had regained the weight they’d lost by the time they were ready for surgery.

*“I have put on approximately ten kilos in the last seven months, waiting. So, there is no help that I’ve received between then and now in order to say, oh, keep your weight down”* (Patient P2).

### 4. The future of the pathway

Nearly all professional participants emphasised the importance of health optimisation and perceived that the policy should be revisited and redesigned for wider implementation across elective surgical referrals with work to improve the engagement of patients with the support on offer.

*“It’s amazing how many people at the start of it are convinced they need surgery and then at the end, right actually, yeah, I think we’re doing okay.”* (Provider Pr6).

*“By far what we’ve found is the people who have engaged and gotten some extra support have done a lot better with their outcomes… People who do get support from Healthy Lifestyles do a lot better than the people who decide to do it themselves.”* (Provider Pr10).

All patients interviewed supported the continuation of the policy within hip and knee surgery and could not offer any circumstances under which they thought it would need to be discontinued. The benefits they cited were numerous. Some felt it offered a feeling of control and the chance for reflection.

*“I don’t think that waiting is a bad thing. It gives you chance to review the situation (…) you could just sort of go on down it without much thought.”* (Patient P5).

Others focused on the benefit of feeling fitter for surgery or avoiding the need for surgery.

*“Well if it leads to having the operation and it’s more successful (…) then that’s a success isn’t it for the pathway and I think for me as well it was a success because it did such a good job I didn’t need an operation.”* (Patient P7).

Improvements to their overall wellbeing and a motivation to gain long term advantages were also described as likely benefits.

*“it would be remiss of me not to make sure these joints have the best chance for a long life and that’s part of weight loss.”* (Patient P4).

Both professionals and patients advocated for personalised support options to boost the chances of patients engaging with the interventions on offer.

*“We need to ensure (…) that they are going to follow it through (…) so that it’s not setting them up with a programme to fail; it’s setting them up with a programme that we know is going to help them and long term as well.”* (Provider Pr13).

#### Considerations for wider roll-out

While the implementation of health optimisation within the hip and knee pathway was deemed a success, the roll-out of the health optimisation policy to gynaecology and general surgical referrals had failed to attract enough clinician support and was eventually reversed. Professional participants reflected on the reasons for this: the wider roll out required GPs to inform the patient they would undergo a delay despite also having agreed that the patient needed a surgical referral, which undermined the GP role as patient advocate. It also required a ‘bureaucratically jumbled process’ where GPs were required to recall the patient to send the surgical referral after the 12 weeks for HO had elapsed. GPs could not refer to surgery and HO simultaneously due to the risk of breaching the ‘referral to treatment time’. In contrast, HO on the hip and knee pathway allowed GPs to ‘complete’ a referral to the hip and knee team with no requirement to ‘hold’ or call back the patient after 12 weeks.

*“it was very uncomfortable leaving this door open for three months and saying, ‘Well, we’ll do the referral. Then we’ll have to get you back and then we’ll have to weigh you’ and then when am I going to write the referral?”* (Managerial M3).

There was also a perception that weight loss offered symptom relief in osteoarthritis but would not offer symptom relief for many other surgical conditions, and that deferral of joint replacement can be desirable to prevent revision surgery in the future. In contrast, deferral of gynaecological or general surgical procedures were not perceived to be of any benefit to the patient.

*“they said, …. If we can put it [hip replacement] off for a couple of years … maybe [it will] see you out.“ (*Patient P2).

## Discussion

Health optimisation interventions were acceptable to the participants in our sample in the orthopaedic pre-surgical context and offer a chance to support both short term pre-surgical weight loss/smoking cessation and longer-term sustained changes to lifestyle intentions post-surgery. Patients in this study indicated an expectation that their primary care clinicians raise weight management and smoking cessation recommendations with them at the point of surgical referral and did not find this problematic, with some stating they would welcome the offer of interventions to support them at this point.

 Health optimisation was successfully implemented as part of the locality’s hip and knee pathway but had failed to be implemented for a wider range of conditions due to poor referral rates. It was suggested that HO during the ‘usual waiting time’ for surgical referral may be of greatest acceptability to professionals and patients. The success of HO was thought likely to be contingent on clinician support for the programmes and how well they are able to communicate the programme’s aim and nature to patients; the quality of the explanation received and understanding gained by patients interviewed in this study was generally low.

Patients and professionals supported the continuation of the HO pathway studied here and recognised likely health and wellbeing benefits for a majority of patients whilst also showing an acceptance of the perceived NHS resource considerations of such policies. A need to personalise the support available to patients to avoid creating inequalities was highlighted.

### Relation to other studies

The related paper from the study of this HO policy [11] details the evaluation’s quantitative findings. These findings show that 37 % of hip and knee patients in the period studied were eligible for health optimisation, mostly due to their BMI. Only 28 % accepted a referral for professional support and they had a larger average reduction in BMI than those who did not accept referral. Data were not collected on whether the others may have attempted self-management or may not have engaged with lifestyle change. Our qualitative findings highlight the importance of identifying patient success in behaviour change and symptom reduction, understanding the reasons for poor and unequal patient engagement with HO programmes and ensuring that programmes are examined for unintended consequences. The potential worth of HO policies has been indicated by the PREP-WELL project; a 6–8 week pre-surgical programme including smoking cessation and weight management support [13]. More than 70 % (53 of 75) of patients gained clinically meaningful improvements in their health status and quality of life through participation.

While there is an acceptance that it would be preferable for patients to be non-smokers with a healthy BMI at the point of their hip or knee surgery [14], literature on the use of thresholds such as BMI or smoking status to limit access to surgery highlights that this practice is neither well-supported from a cost-saving nor an ethical perspective [3, 5, 15]. Studies of patients’ responses to rationing of healthcare highlight the disparities between the decisions they report they would make in theory and their attitude when faced with a direct impact on their personal receipt of care [16]. Our study patient participants faced a prolonged surgical pathway and therefore offer valuable insight given that they were in support of continuation of the policy. With patient reactions to rationing affected by the attitude of the clinical team providing the care, training clinicians in relevant communication skills is valuable [16].

A recent UK study using patient questionnaires concluded that there is substantial patient desire to modify behaviours for peri-operative benefit and identified the need for structured pre‐operative support, but did not offer any qualitative data collection from the participants [17]. The findings of a qualitative study that behaviour change interventions were perceived by patients as appropriate and helpful during routine GP consultations, particularly where behaviour change could have a positive effect on long-term conditions. This aligns with the way in which our participants highlighted the vital role of the GP in introducing and endorsing the pathway in this study to their patients [18]. A review of the role of psychological factors in prehabilitation for surgical interventions recommends that interventions are developed with an understanding of patient needs and preferences and potential barriers to engagement to produce person-centred approaches [19]. Our findings suggest that careful consideration of the framing of a health optimisation programme, and recognition that behaviour change for lifestyle-related risk factors is not straightforward, is of importance in the communication between clinicians and patients.

### Strengths and limitations

The main strength of this study is that it offers insight into the experiences and perspectives of patients and other key stakeholders who have experienced the creation and successful implementation of a HO programme. In-depth interviews with the participants offered much richer information than the feedback questionnaires used in other settings [13]. The study design and nature of the interview topic guides were well-informed by the multidisciplinary study steering group and patient and public involvement, ensuring that we addressed multiple viewpoints and priority issues in our investigation.

A limitation of the study is that participants may have been more likely to participate if they held particularly strong views on the policy. The COVID-19 pandemic necessitated a halt to recruitment after four months, due to social distancing regulations and the suspension of patient contact with the hip and knee team. This curtailment of our data collection prevented the inclusion of a larger representation of GP participants and patients who were smokers which would have benefitted the sample. Further views may emerge from investigation of similar settings with different health optimisation programmes in place.

### Implications for policy makers and clinicians

The results of this study can be used to inform the introduction and content of HO policies in other health care systems.

Recommendations:

1. Ensure that the communication of the intent and nature of the health optimisation pathway is positive and individualised at the point of initial patient contact with primary care. Clinicians should have confidence that most patients are likely to expect and welcome an offer of support for weight loss or smoking cessation in this context and that patient awareness of hoped for reductions in need for surgery need not be problematic.
2. Address patient engagement with health optimisation programmes by widening the available choice of support on offer and including qualitative evaluation of new programmes. The group-based interventions used here may be acceptable to many but those wanting an individualised approach have fewer options and particular consideration should be given to providing interventions matched to groups experiencing health inequalities.

## Conclusions

Both clinicians and patients who had used the health optimisation pathway for hip and knee surgery found it to be generally positive, and they supported the continuation in use of such policies in this setting. The COVID-19 pandemic led to the suspension of much elective surgery worldwide and as a result the number of patients awaiting such surgery is higher than ever [10]. As such, effective pre-surgical health optimisation would be of significant benefit to the NHS and other healthcare settings and may reduce the requirement for surgery. This work can guide the development of future policies and their implementation and should help to inform the consideration of mitigating actions to reduce the potential inequalities introduced or exacerbated by such pathways.

## Data Availability

The datasets used and/or analysed during the current study are not publicly available due to the need to maintain privacy of the participants but are available from the corresponding author on reasonable request.

## Abbreviations

BMI: body mass index
CCG: clinical commissioning group
GP: general practitioner
HO: health optimisation
NHS: National Health Service
PPI: patient and public involvement
UK: United Kingdom
